# Venous thromboembolism prophylaxis in patients undergoing knee replacements: comparison of real-world outcomes

**DOI:** 10.1007/s11096-020-01173-3

**Published:** 2020-10-18

**Authors:** Syed Shahzad Hasan, Wendy Sunter, Nadia Ahmed, Dalia Dawoud, Syed Tabish Razi Zaidi

**Affiliations:** 1 Department of Pharmacy, University of Huddersfield, Huddersfield, UK; 2 School of Biomedical Sciences and Pharmacy, University of Newcastle, Newcastle, Australia; 3 Calderdale and Huddersfield Anticoagulant Clinic, Calderdale and Huddersfield NHS Foundation Trust, Huddersfield, UK; 4 Department of Clinical Pharmacy, Faculty of Pharmacy, Cairo University, Cairo, Egypt; 5 School of Healthcare, University of Leeds, Leeds, UK; 6 Leeds Teaching Hospitals NHS Trust, Leeds, UK

**Keywords:** Apixaban, Aspirin, Knee replacement, Prophylaxis, Venous thromboembolism

## Abstract

*Background* Increasing evidence for the use of the aspirin in patients undergoing an orthopaedic surgery for venous thromboembolism prophylaxis has led to a change in the national guidelines substituting anticoagulants with aspirin. Little is known about the impact of such substitution on real-world outcomes from clinical practice. *Objective* The study was designed to examine clinical outcomes associated with the use of aspirin and apixaban. *Setting* Two large-scale general hospitals in West Yorkshire region of England. *Method* A 1-year observational study among adults who underwent elective knee replacements and received venous thromboembolism prophylaxis within the first 14 days post replacements. *Main outcome measure* The incidence of postoperative venous thromboembolism, leaking wounds during the hospital stay, and 30-day any readmission for the two drugs. *Results* A total of 420 patients were included. There was a significant drop in apixaban prescribing (from 80.37 to 10.51%) and increase in aspirin use (from 19.02 to 81.71%) after the implementation of the revised guidelines. There were 52 (12.38%) cases of leaking wound, 16 (3.81%) cases of postoperative venous thromboembolism, 45 (10.71%) cases of 30-day readmission and no case of 30-day major bleeding. The leaking wounds and 30-day readmissions were almost twice more frequent in obese compared to non-obese patients. Multivariate logistic regression found an increased risk of leaking wound with apixaban and postoperative venous thromboembolism and 30-day readmission with aspirin use but the differences were not statistically significant. *Conclusion* The results suggest aspirin to be as effective as apixaban in preventing venous thromboembolism and readmission. Apixaban usage decreased with a corresponding increase in Aspirin use. The impact of obesity and length of hospital stay need further investigations.

## Impacts on practice

- Both aspirin and apixaban have been proven to reduce the risk of venous thromboembolism in patients undergoing orthopaedic surgery in controlled studies but there is limited real-world data exists from routine clinical practice.
- A significant decreased in apixaban usage with a corresponding increase in aspirin use; signalling good adherence to national guidelines.
- Aspirin is safe and can be given to patients undergoing knee replacement surgery to prevent venous thromboembolism.

## Introduction

About 1.2 million knee replacements were performed between 2003 and 2018 in England, Wales, Northern Ireland and Isle of Man [1]. Patients undergoing these procedures are at an increased risk of venous thromboembolism (VTE). Appropriate VTE prophylaxis is an essential component of all major orthopaedic procedures. The incidence of VTE in patients with appropriate VTE prophylaxis is 1.3–10% which drastically increased to 40–60% in those who do not receive such prophylaxis [2]. Despite the use of effective thrombo-prophylactic treatment and adherence to the most appropriate guidelines, 0.5–4.0% of patients still develop symptomatic VTE after hip and knee replacement surgery, with 0.2–1.9% suffering from acute PE [3].

Apixaban has been proven to reduce the risk of VTE in patients undergoing total joint arthroplasty. In 2013, a systematic review and meta-analysis [4], found direct oral anticoagulants (dabigatran, apixaban and rivaroxaban) and standard-dose vitamin k antagonists as superior to aspirin and placebo in lowering the risk of recurrent VTE. They also suggested apixaban as the best drug for reducing bleeding risk among the new oral anticoagulants. In 2015, a network meta-analysis [5], supported the ACCP guidelines at the time, found that all three DOACs (apixaban, dabigatran, rivaroxaban) were found to reduce the risk of VTE at similar rates when compared to a placebo, except aspirin. Apixaban had a significantly lower bleeding risk than the other oral anticoagulants. In 2016, another systemic review and network meta-analysis [6] found apixaban as the only DOAC which had a significantly lower risk of bleeding (non-major and major) than warfarin and other DOACs. The risk of DVT, non-fatal PE and VTE related deaths were similar across the different treatments, except the rate of DVT which was significantly lower for all DOACs compared to aspirin [6].

Recent trials have shown that aspirin is as effective as anticoagulants in preventing VTE, with a lower incidence of wound leaking, post-operative bleeding, hospital readmission, and mortality [7, 8]. Simes et al. [8] performed the combined analysis of WARFASA and ASPIRE trials (n = 1224) and found that aspirin reduces the risk of recurrent VTE by 40% and the incidence of major bleeding was similar (0.5%/year) when compared to placebo (0.4%/year) [8]. A systematic review and meta-analysis (2014) on randomized trials comparing aspirin to anticoagulants following major orthopaedic arthroplasties found aspirin as effective as anticoagulants in reducing the rates of VTE with lower bleeding risk [9]. Another systematic review [10] found similar rates of VTE with aspirin when compared to other prophylactic agents in total hip or knee replacements in a previous meta-analysis of clinical trials [11]. The incidence of major bleeding episodes was also found to be low, although the extent of bleeding complications was unclear [10]. Despite the availability of good quality evidence from systematic reviews and meta-analyses in support of aspirin for the prevention of VTE in orthopaedic surgery, there is a scarcity of data about the effectiveness of aspirin using real-life data.

The National Institute of Health and Care Excellence (NICE) in 2018, revised its guidelines for VTE prophylaxis for patients undergoing orthopaedic surgery [12]. The guideline recommends offering prophylaxis for patients whose risk of VTE outweighs the risk of bleeding. Aspirin or low molecular weight heparin (LMWH) are recommended to be used for 14 days as pharmacological prophylaxis [13]. In the economic modelling, apixaban or dabigatran were found to be less cost-effective compared to aspirin and LMWH [14].

### Aim of the study

This study aimed to examine clinical outcomes associated with the use of aspirin and apixaban. We compared the two drugs for the incidence of postoperative VTE, leaking wounds, and readmission (30-day and 6-month) due to bleeding. Besides, we investigated the association of obesity and length of hospital stay with clinical outcomes.

### Ethics approval

Ethics approval was attained from the University of Huddersfield School of Applied Sciences Research Integrity and Ethics Committee (Project ID: SAS-SREIC 4. 1. 19-8). Permission to access the hospital facilities and collect patient data was also obtained from CHFT hospital before beginning the data collection process. To maintain patient confidentiality at all times, each case was given a unique code so that no personal details were taken out of the hospital. As the study was conducted from medical records, individual informed consent was not applicable.

## Methods

This was a 1-year observational study among patients receiving VTE prophylaxis following elective Primary Total Prosthetic Replacement of Knee (PTPRK) at Calderdale and Huddersfield NHS Foundation Trust (CHFT) hospitals. This study was carried out in awake of the implementation of revised guidelines recommending the use of aspirin instead of the previously used apixaban. The study has been designed to provide a comparison of clinical outcomes seen in orthopaedic patients at CHFT with the use of each of the two drugs.

Both Huddersfield Royal Infirmary and Calderdale Royal Hospital are located in West Yorkshire, England. Both sites offer clinics and pre-assessment, but operating theatres and elective orthopaedic wards are based at the Calderdale site. Patients are transferred from the recovery area in the theatres to the orthopaedic wards, where they are closely monitored throughout their hospital stay. The first dose of VTE medication is administered on the first day after surgery and the aim is to discharge patients 2–3 days post-surgery providing there are no concerns [15].

The study included all patients over the age of 18 who had undergone PTPRK at CHFT between August 1, 2018, and July 31, 2019: between August and October 2018 to capture data on apixaban (a drug of choice) and between November and July 2019 to capture data on aspirin (a drug of choice). Revised NICE guidelines for antithrombotic prescribing, NG89, were introduced in March 2018 by NHS England. New hospital guidelines were implemented later in the same year (November 2018) in accordance to NG89, encouraging doctors at CHFT to prescribe Aspirin 75 mg daily (may be increased to 150 mg daily for higher-risk patients, decided on a clinical basis) as first-line VTE prophylaxis in elective knee surgery patients. Apixaban 2.5 mg twice daily was recommended for use as second-line treatment in specific patient groups. Exceptions to using Aspirin include such as previous VTE or other additional risks of VTE. The data were collected regardless of gender or ethnic background. However, those patients who were deceased or had incomplete medical records were excluded from the study.

VTE prophylaxis used such as the name, duration, number of administered doses, and number of missed doses with reasons, BMI, length of hospital stay were collected from medical records. All patients who were operated on during the study period and met the inclusion criteria were included. The required information was obtained by navigating through the different sections of the electronic medical records system.

Any leaking wounds (at the surgical site) during the hospital stay, post-operative VTE (any event of VTE within 30 days of surgery), any readmission within 30 days (any readmission within 30 days of surgery), 30-day any major or clinically relevant non-major bleeding and any changes to VTE prophylaxis were recorded.

All data were collated onto a spreadsheet document from which it was analysed using SPSS version 25 with a significance level of 0.05. Patient age was categorised into three categories—middle-aged, older adults and elderly. BMI was categorised into a healthy weight, overweight, obesity I, Obesity II and Obesity III using the adult international BMI classification [16]. Categorical variables such as VTE medication and any re-admission were compared using the Chi-squared test or Fisher’s exact test. Differences between the independent groups (apixaban and aspirin) concerning clinical outcomes were determined using independent t-test and Chi-squared test or Fisher’s exact test. Unadjusted and adjusted odds ratios using logistic regression were used to assess the association between VTE medicines or VTE prophylaxis guideline and post-surgery outcomes. The association between the choice of VTE medication and the clinical outcomes was then further analysed using logistic regression.

## Results

**Fig. 1:**
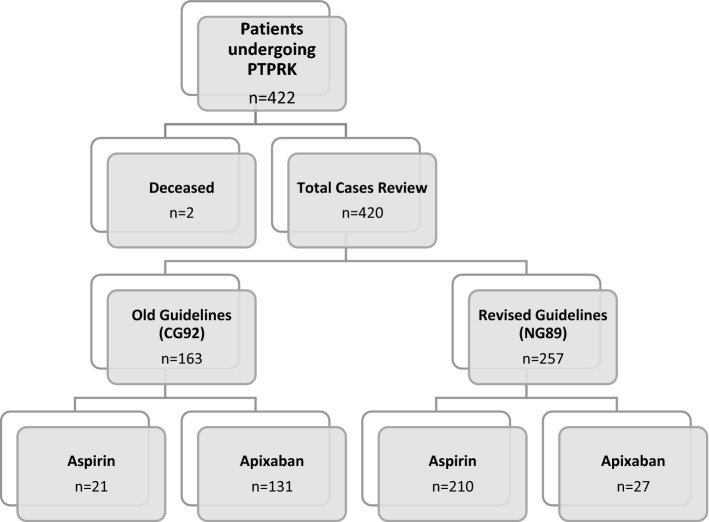
Information regarding patients and VTE prophylaxis included for data analysis during the study (*PTPRK* = *Primary Total Prosthetic Replacement of Knee*)

A total of 420 patients who underwent knee replacement surgery at CHFT hospitals were included. Of the total, 163 patients were operated between 1 August 2018 and 31 October 2018 and 257 were operated between 1 November 2018 and 31 July 2019 (Fig. 1).

**Fig. 2:**
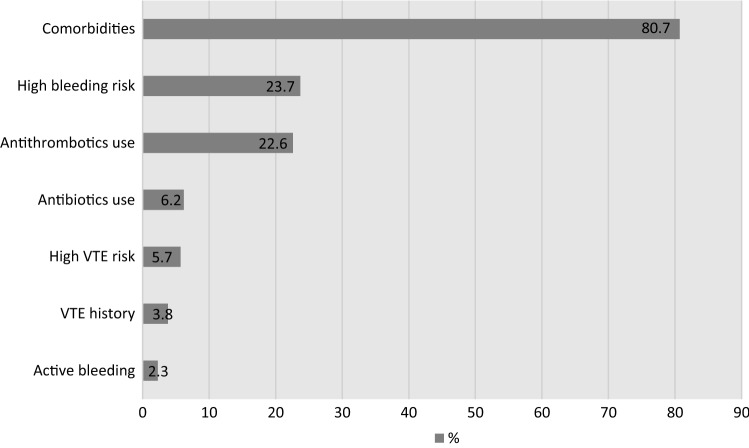
Pre-op medications use, VTE and bleeding risks and comorbidities

Figure 2 presents the pre-operation variables. More than two-thirds of the patients had comorbid conditions (80.7%). High bleeding risk (23.7%) was more prevalent than high VTE risk (5.7%). Only 23% of the patients were on antithrombotic agents (including aspirin).

The age of patients in the study varied between 41 and 89 years (Table 1). There was an even spread between the lowest and highest age, with an overall average of 68.99. Only 9% of patients were of a healthy weight whereas the majority were categorised as obese (66.0%). Only a few patients in the study were taking additional medications before surgery.

**Table 1:** Characteristics of study participants and VTE prophylaxis

Item	Value	Minimum	Maximum
Age (mean ± SD)	68.80 ± 9.73	41	92
BMI (mean ± SD)	31.31 ± 6.39	18.71	78.00
BMI categories 1 [n (%)]			
18.5–24.9 (healthy weight)	38 (9.0)		
25–29.9 (overweight)	107 (25.4)		
30–34.9 (obesity I)	110 (26.1)		
35–39.9 (obesity II)	49 (11.6)		
40 or more (obesity III)	117 (27.8)		
BMI categories 2 [n (%)]			
< 30 (non-obese)	143 (34.0)		
30 or more (obese)	277 (66.0)		
Length of stay (mean ± SD)	3.33 ± 3.10	0	38
0–1 day	40 (9.50)		
2–3 days	201 (47.70)		
> 3 days	180 (42.80)		
No. of days of treatment (mean ± SD)	13.90 ± 1.51		
No of doses administered at hospital (mean ± SD)	3.82 ± 2.62		
No of missed doses (mean ± SD)	0.17 ± 0.52		
VTE medication [n (%)]			
Warfarin	12 (2.90)		
Aspirin 75 mg	156 (37.10)		
Aspirin 150 mg	76 (18.10)		
Apixaban 2.5 mg	158 (37.9)		
LMWH	10 (2.4)		
Rivaroxaban	5 (1.2)		
VTE prophylaxis based on [n (%)]			
Old guideline (CG92)	163 (38.8)		
New guideline (NG89)	257 (61.2)		
VTE prophylaxis according to guidelines [n (%)]			
Yes	322 (76.7)		
No	19 (4.5)		
No information	79 (18.8)		

There was a significant drop from 80.37 (131/163) to 10.51% (27/257) in apixaban prescribing post-October 2018 whereas aspirin use was increased from 19.02 (31/163) to 81.71% (210/257) after implementation of revised guidelines. More than two-thirds of the patients received VTE prophylaxis according to the guidelines. For both drugs, the mean duration of VTE prophylaxis medication was about 14 days which was according to the guidelines. The mean length of hospital stay for patients on these drugs was close to 3 days.

In the 1-year study period, there were 52 (12.38%) cases of leaking wound, 16 (3.81%) cases of postoperative VTE, 45 (10.71%) cases of 30-day readmission, 7 cases of 30-day non-major bleeding, and no case of 30-day major bleeding post knee replacement surgery (Table 2). Amongst patients with leaking wound, 6.0% were on apixaban and 5.3% were on aspirin (*p* = 0.325). Of 25 cases of missed doses, 68% of patients were on apixaban whilst the remaining 32% were on aspirin (*p* = 0.522). The VTE incidence was not significantly different between aspirin and apixaban (2.4% vs. 0.7%, *p* = 0.152).

**Table 2:** Clinical outcomes by VTE prophylaxis medicines

Item	Leaking wound (52 cases) n (%)	Postoperative VTE (16 cases) n (%)	30-day any readmission (45 cases) n (%)
BMI categories 2	*n* = 418	*n* = 417	*n* = 419
< 30 (non-obese)	19 (4.5)	7 (1.7)	15 (3.6)*
30 or more (obese)	33 (7.9)	9 (2.2)	30 (7.2)
Length of stay	*n* = 418	*n* = 417	*n* = 419
0–1 day	1 (0.2)	0 (0.0)	2 (0.5)
2–3 days	24 (5.7)	8 (1.9)	23 (5.5)
> 3 days	27 (6.5)	8 (1.9)	20 (4.8)
VTE medicines	*n* = 415	*n* = 414	*n* = 416
Aspirin (75 mg and 150 mg)	22 (5.3)	10 (2.4)	22 (5.3)
Apixaban (2.5 mg)	25 (6.0)	3 (0.7)	18 (4.3)
Other (warfarin, rivaroxaban, dalteparin)	4 (1.0)	2 (0.5)	4 (1.0)

*p < 0.05

The cases of leaking wound during hospital stay (7.9% vs. 4.5%, *p* = 0.220) and 30-day readmission (7.2% vs. 3.6%, *p* = 0.05) were twice more frequent in obese patients than in non-obese patients. Patients who were reported with leaking wound stayed longer in hospital. However, this observation was not statistically significant.

The results from the logistics regression analysis looking at the association between the choice of VTE prophylaxis medication (aspirin or apixaban) and three key clinical outcomes can be seen in Table 3. We have excluded 30-day non-major bleeding from logistic regression due to a small number of cases. Both unadjusted and adjusted regression models found an increased risk of leaking wound with apixaban and post-operative VTE and 30-day readmission with aspirin use but the differences were not statistically significant. Similarly, there were increased odds of adverse clinical outcomes with obesity but again associations were not statistically significant. The only statistically significant association was between the length of stay and 30-day readmission (OR 1.31, 95% CI 1.04–1.63).

**Table 3:** Odds ratio (95% CI) of leaking wounds, readmission, and 30-day minor bleeding, according to VTE prophylaxis medicines

Item	No referent	Leaking wounds (yes)		Postoperative VTE (yes)		30-day any readmission (yes)		
		Unadjusted OR (95% CI)	Adjusted OR (95% CI)	Unadjusted OR (95% CI)	Adjusted OR (95% CI)		Unadjusted OR (95% CI)	Adjusted OR (95% CI)
VTE prophylaxis								
Apixaban	1.0	1.0	1.0	1.0	1.0		1.0	1.0
Aspirin		0.56 (0.31–1.04)	0.74 (0.25–2.20)	2.31 (0.63–8.52)	2.62 (0.05–155.58)		0.82 (0.42–1.58)	1.52 (0.40–6.66)
BMI category								
< 30 (non-obese)	1.0	1.0	1.0	1.0	1.0		1.0	1.0
30 or more (obese)		0.87 (0.47–1.59)	1.53 (0.52–4.48)	0.65 (0.24–1.79)	2.89 (0.27–31.03)		1.03 (0.53–1.98)	0.73 (0.19–2.85)
BMI	1.0	1.03 (0.99–1.08)	1.05 (0.99–1.11)	1.00 (0.92 0 1.09)	1.08 (0.85–1.19)		1.02 (0.97–1.07)	1.03 (0.96–1.04)
LOS	1.0	1.10 (0.99–1.22)	1.21 (0.97–1.48)	1.12 (0.97–1.29)	1.10 (0.63–1.09)		1.09 (0.98–1.21)	1.31 (1.04–1.63)*

Adjusted for age, sex, BMI, and length of hospital stay (LOS)

*OR *odds ratios

## Discussion

The present study reported real-life clinical practice data from hospitals in West Yorkshire, England following the change in the NICE recommendation to use aspirin instead of anticoagulants for VTE prophylaxis post orthopaedic surgery. Following the introduction of revised guidance based on NICE guidelines encouraging the use of aspirin at the study hospitals, we observed adherence of over 80% with the guidelines within the first 6 months. This suggests that prescribers were aware of the changes and remained compliant with the revised guidelines. We compared the incidence of postoperative VTE, leaking wounds, and 30-day readmission in patients who received aspirin and apixaban for VTE prophylaxis. In the 1-year study period, leaking wound during hospital stay was the most common clinical event (52 cases) followed by 30-day readmission (45 cases), and post-operative VTE (16 cases).

Wound complications such as leaking can significantly affect the outcome of orthopaedic surgery and can be an early indication of periprosthetic joint infection that may lead to skin necrosis, loss of prosthesis and amputation in severe cases [17]. The study found a total of 52 (12.4%) wound leaking cases amongst knee elective surgery patients at CHFT. There have been between 0.2% and 21% reports of wound leaking after total joint arthroplasty, with a higher incidence rate after revision surgery [18]. We did not find a statistically significant association between the choice of drug prophylaxis and wound leaking during the hospital stay. The risk of leaking wound with use of these drugs was low. Previously, both aspirin and apixaban have been found to increase the risk of wound complications [6]. In comparison to no pharmacological prophylaxis, a study found aspirin to significantly increase the risk of wound leaking and concluded there was a need for careful assessment of risk versus benefit when prescribing VTE prophylaxis [19]. Similarly, another study found that patients who received factor Xa inhibitors such as apixaban for VTE prophylaxis after primary total joint arthroplasty had a significantly higher rate of wound complications and bleeding [20].

Duration of hospital stay is another factor that could be influenced by wound leaking or thromboembolic event. Prolonged hospital stay not only increases the NHS costs but can also be limiting for the patient and family and there is an increased risk of surgical site infections [21]. The mean length of hospital stay for patients on these drugs was significantly different (close to 3 days) in this study. This study found a statistically significant association between length of stay and 30-day readmission. Basques et al. found that the rate of thromboembolic events was higher amongst inpatients than patients who were discharged on the same day after orthopaedic surgery, whilst a higher number of patients required more surgery in those who returned home the same day [22]. This highlights the importance of balancing the risk of VTE with other adverse outcomes such as readmission post-surgery.

There were three times as many VTE incidents for aspirin than apixaban, however, no statistically significant association was found between VTE incidence and choice of the antithrombotic agent after elective knee replacement surgery. Similarly, 30-day readmission was also higher in patients treated with aspirin than apixaban, but the difference was not statistically significant. The readmission cases could be due to wound leaking among other factors. A study in 2013 found that wound complications, wound infection, bleeding and reduced mobility were common reasons for re-admission after total knee arthroplasty [23].

There were no 30-day major bleeding events reported and only 7 (3 in apixaban and 4 in aspirin) cases of 30-day non-major bleeding post total knee replacement surgery. This shows that both drugs are safe in term of bleeding risk. A very small proportion of total knee replacement patients requiring hospitalisation for bleeding were reported in a study but the figure was thought to be an underestimation as bleeding events after initial knee replacement surgery are not always recorded as secondary discharge diagnosis in hospitals [24]. The risk of gastrointestinal bleeding during the initial 6 weeks after total knee replacement surgery was reported to rise by 2.3-fold and the use of a proton pump inhibitor alongside aspirin as VTE prophylaxis was seen to decrease this risk in patients [25]. This explains the suggested use of a proton pump inhibitor with aspirin in patients with gastric irritation or increased risk of GI bleeding. Although anticoagulants such as apixaban are highly effective in the prevention and treatment of VTE, they have also been associated with an increase in the risk of bleeding [6, 26]. However, the risk of haemorrhage is significantly influenced by individual clinical factors such as anaemia, increasing age and renal disease [27]. This shows that the risk varies between patients and is influenced by a combination of many factors.

We did not find any significant increase in wound leaking, the post-operative incidence of VTE, and 30-days readmission rates in obese versus non-obese patients in our study. A meta-analysis of safety and efficacy of direct oral anticoagulants (DOACs) use in major orthopaedic surgery found that the recommended dose of Apixaban is sufficient to provide VTE prophylaxis [28]. We were unable to find any specific study on the use of Aspirin for VTE prophylaxis in obese patients undergoing major orthopaedic surgery, Obesity is considered as an independent risk factor for VTE in patients undergoing major orthopaedic surgery [28]. We were unable to locate any clinical study on aspirin use as VTE prophylaxis in obese patients through a pharmacodynamic study concluded that obese patients may need a higher than the normal dose to achieve the similar antithrombotic effect [29].

Appropriate administration of VTE prophylaxis medication is vital as up to 25 000 deaths out of 60 000 were found to be avoidable with the use of appropriate VTE prevention [21]. Mean duration of 14 days was found for both aspirin and apixaban regimens with no significant difference in mean values. The majority of pharmacological VTE prophylaxis is given for a minimum of 10 to 14 days and up to 35 days [2]. Aspirin and apixaban are recommended as VTE prophylaxis in patients receiving elective knee replacement surgery for 14 days [30]. This suggests that patients at CHFT received an appropriate duration of pharmacological prophylaxis in accordance with guidelines.

Various limitations within the study have been recognised. The risk of selection bias cannot be ruled out. The uneven number of participants for each drug limited the quality of comparison which could be made between the two groups. Using an equal number of participants on aspirin and apixaban would have provided better results and conclusion. As we have mentioned above a very small proportion of patients associated with bleeding were reported in a study could be a result of underestimation. We have limited information about pre and post-surgical medications, many patients using continued aspirin therapy for other indications after surgery. Due to the limitation of study design employed in this study, it was difficult to perform cause and effect analysis or explain the causal pathway for various associations discussed in our article. Furthermore, the study relied heavily on the accuracy of patient records, so if there was any underreporting, this will have influenced the results.

## Conclusions

The study has successfully provided a comparison between clinical outcomes seen in orthopaedic patients undergoing elective knee replacement surgery and receiving apixaban and aspirin using real-world data. Aspirin is the most commonly prescribed antithrombotic agent for VTE prophylaxis. The use of apixaban dropped whilst aspirin prescribing increased after revised guidelines were implemented. The average duration of VTE prophylaxis following elective knee surgery for both aspirin and apixaban was 14 days. No statistically significant association between the choice of pharmacological VTE prophylaxis (aspirin or apixaban) and any of the clinical outcomes was found in the study.
